# From Linkage Studies to Epigenetics: What We Know and What We Need to Know in the Neurobiology of Schizophrenia

**DOI:** 10.3389/fnins.2016.00202

**Published:** 2016-05-11

**Authors:** Ariel Cariaga-Martinez, Jerónimo Saiz-Ruiz, Raúl Alelú-Paz

**Affiliations:** ^1^Laboratory for Neuroscience of Mental Disorders Elena Pessino, Department of Medicine and Medical Specialties, School of Medicine, Alcalá UniversityMadrid, Spain; ^2^Department of Psychiatry, Ramón y Cajal Hospital, IRYCISMadrid, Spain; ^3^Centro de Investigación Biomédica en Red de Salud Mental (CIBERSAM)Madrid, Spain

**Keywords:** epigenetics, schizophrenia, neuroscience, DNA methylation, psychiatric diagnose

## Abstract

Schizophrenia is a complex psychiatric disorder characterized by the presence of positive, negative, and cognitive symptoms that lacks a unifying neuropathology. In the present paper, we will review the current understanding of molecular dysregulation in schizophrenia, including genetic and epigenetic studies. In relation to the latter, basic research suggests that normal cognition is regulated by epigenetic mechanisms and its dysfunction occurs upon epigenetic misregulation, providing new insights into missing heritability of complex psychiatric diseases, referring to the discrepancy between epidemiological heritability and the proportion of phenotypic variation explained by DNA sequence difference. In schizophrenia the absence of consistently replicated genetic effects together with evidence for lasting changes in gene expression after environmental exposures suggest a role of epigenetic mechanisms. In this review we will focus on epigenetic modifications as a key mechanism through which environmental factors interact with individual's genetic constitution to affect risk of psychotic conditions throughout life.

## Introduction

We define schizophrenia as a complex psychiatric illness characterized by the presence of positive, negative, and cognitive symptoms that affect multiple aspects of mental activity, including perception, thought, attention, memory, and emotion. The age at onset is typically in adolescence or early adulthood, with a median lifetime prevalence of 4.0 per 1000 and a morbid risk of 7.2 per 1000. The ratio of observed deaths to expected deaths for all-cause mortality is 2.6 for patients with schizophrenia compared to the general population. The concordance rates of schizophrenia for monozygotic twins have been estimates to be about 40–50%, and heritability around 80% (Gejman et al., 2010). To date, this disorder lacks a unique and defining pathophysiology, despite the abundance of basic and clinical research. In fact, the massive quantity of information generated during the last decades has been seen as an indicator of uncertainty and confusion in searching for pathognomonic signs or symptoms, more than a useful tool (Maj, 2011).

However, several research approaches give reason for more optimism, focusing on a link that remains undefined: the neurobiological and behavioral basis of the disease that correlate with the patient's clinical features (Tandon et al., 2009). In this regard, the development of the Reseach Domain Criteria (RDoC) represents a new and integrative way to classify mental disorders based on dimensions of observable behavior and neurobiological measures (Cuthbert and Insel, 2013). The proposal is, at least, ambitious; RDoC implies both conceptual and practical differences compared to traditional classification systems, that is, DSM or ICD. First, RDoC goes from pathophysiology to clinical aspects, including a dimensional approach that encompass the full range of variation, from normal to abnormal, trying not to focus on at one end or the other of the continuum rather than focus on those zones of very mild or transient psychopathology. This approach could help us to achieve a more precise specification of the genetic, epigenetic, molecular and cellular aspects of mental disorders.

Here we will briefly review the main findings in studies of linkage and association, genome wide association, quantitative trait loci, endophenotypes, and structural and functional neuroanatomy, describing advantages and possible limitations of each one. Finally, we will focus on the role of epigenetics as a more recent scientific approach that may help us to understand, from an RDoC paradigm, the complex ways in which nature interacts with nurture to sometimes produce a severe mental illness.

## Linkage and association studies

Parametric and nonparametric linkage analysis have been applied with success in studies of Mendelian disorders, which are characterized by the presence of a single major locus with rare highly penetrant alleles (Badano and Katsanis, 2002). In complex non-Mendelian illnesses, such as schizophrenia, a genetic model for linkage studies is difficult to establish (Risch, 1990), and former research attempts to identify different schizophrenia susceptibility loci showed poor replication (i.e., 6p24-22, 6q25, 6q23, 10q24, and 17q21)(Straub et al., 1995; Lindholm et al., 2001; Lerer et al., 2003; Williams et al., 2003; Escamilla et al., 2009). Even more, a recent meta-analysis (Walters et al., 2014) demonstrated, after computational data cleaning, substantial differences in results reported in older linkage studies, underscoring the limitations of those previous analysis. Why linkage analysis fails to describe schizophrenia susceptibility genes? Although at the beginning the results obtained in other complex diseases—such as breast cancer or familiar Alzheimer—led to believe that linkage approach could be a fruitful strategy to aid in first identifying genetic and then other etiological factors important in the disorder (Goate et al., 1991), as we mentioned above, linkage has proven to be a method of immense power for Mendelian disorders that differs from schizophrenia in critical ways which make successful much more difficult. The former have full penetrance, the manifestation of their typical symptoms is due to the disease mutation, environment has no incidence in the disease development, exists a clear distinction between affected and non-affected individuals and the same gene is responsible for all the cases of illness in a pedigree. On the contrary, in schizophrenia genes have reduced penetrance, symptoms can be produced by different conditions, environmental factors are critical to account for observed patterns of risk and, finally, does not exist a clear distinction between affected and non-affected individuals (Riley, 2004).

A more powerful technique than linkage analysis are association studies (Risch and Merikangas, 1996), that test differences in allele frequency between cases (individuals with schizophrenia) and control subjects. Unlike linkage, association studies have a higher spatial resolution and sufficient power to detect common genetic variants (Mantripragada et al., 2010).

To its last updated version (2011) the SZGene database (www.szgene.org) collect 1727 association studies, taken from thousands of those published in specialized literature. Meta-analysis from this database identifies 43 candidate genetic variants (“top candidates”), highly associated to schizophrenia phenotypes (Allen et al., 2008). Also, Shi et al. (Shi et al., 2008) selected association studies for 12 of these “top” candidates and its meta-analysis showed significant allelic associations across different populations in genes involved in the metabolism of key neurotransmitters (e.g., *DAO, DRD4, PPP3CC*, serotonin transporter *SLC6A4*) as well as genes related to DNA methylation (e.g., *MTHFR*), apoptosis and neurodevelopment (e.g., *TP53*).

Other studies also found genes involved in the regulation of neurotransmitters implicated in the disorder, such as *COMT* (Chen et al., 2004; Shifman et al., 2004), *DTNBP1* (Straub et al., 2002; Maher et al., 2010), or *RGS4* (Chowdari et al., 2002; Talkowski et al., 2006) or aspects of neural development such as *NRG1* (Stefansson et al., 2002; Munafo et al., 2008) or *DISC1* (Pletnikov et al., 2008; Schumacher et al., 2009).

This approach has important advantages and disadvantages when compared to linkage: although in association studies is possible to study individual patients, the regions of the genome analyzed are smaller than linkage studies, that means that the data obtained must be used for the assessment of candidate genes or regions only. Moreover, association studies can lead to false positives which has a direct incidence in the reliability of the technique and the lack of replication between independent studies (Riley, 2004; Sullivan, 2007).

## Genome wide association studies (GWAS)

According to NIH guidelines (National Health Institute–United States), GWAS is defined as any study of genetic variation across the entire human genome that is designed to identify genetic associations with observable traits (such as blood pressure or weight), or the presence or absence of a disease or condition (Health, 2008). Likened to a survey of the entire human genome for regions that are correlated with disease severity or onset, GWAS assumes that any region across the genome can be a focus for influencing phenotypic variation (Hirschhorn and Daly, 2005; Wang et al., 2005; Girard et al., 2011; Lee et al., 2012) and represents a powerful alternative to the aforementioned analyses given the possibility to study single nucleotide polymorphisms (SNPs) and copy number variants (CNV). As we indicate on Table 1, several groups have employed this methodology (Mah et al., 2006; Lencz et al., 2007; O'Donovan et al., 2008; Shifman et al., 2008; Sullivan et al., 2008; Kirov et al., 2009; Liu et al., 2009; Need et al., 2009; Purcell et al., 2009; Shi et al., 2009, 2011; Stefansson et al., 2009; Athanasiu et al., 2010; Wang et al., 2010; Chen et al., 2011; O'Dushlaine et al., 2011; Ripke et al., 2011; Williams et al., 2011; Yue et al., 2011; Liou et al., 2012) implicating a number of SNPs and various susceptibility loci for the disorder, such as *RELN* (rs7341475 on chromosome 7q22), *RBP1* (rs893703 on chromosome 3q23), *PLXNA2* (rs752016 on chromosome 1q32), *ZNF804A* (rs1344706 on chromosome 2q32.1), *NRGN* (rs12807809 on chromosome 11q24), or *TCF4* (rs9960767 on chromosome 18q21) and chromosome regions 1q32.2, 5q33.2, and 8p21-22, among others, as well as deletions and/or duplications in several chromosomal regions (Lee et al., 2012). Even more, an entire researcher confederation, the Psychiatric Genetic Consortium (PGC), is actively working in genetic data sharing, meta-analysis and data cleaning and organization from GWAS, leading to an important body of information in schizophrenia genetics (Schizophrenia Psychiatric Genome-Wide Association Study, 2011; Schizophrenia Working Group of the Psychiatric Genomics, 2014; Rees et al., 2015; Takahashi et al., 2015). Efforts from PGC in finding risk or susceptibility loci were recently successful by reporting an association of schizophrenia risk with genetic markers across the major histocompatibility complex (MHC). Data from the Schizophrenia Working Group of the PGC found that SNPs in C4 alleles affect its expression in the brain, leading to putative synapse elimination in schizophrenic patients (Sekar et al., 2016).

**Table 1:** **Summary of the most relevant findings in GWAS studies in schizophrenia**.

**Number of Schizophrenic Samples**	**Type of Samples**	**Genes of Interest**	**Chromosome/Locus**	**References**
320	Peripheral blood DNA	*PLXNA2*	1q32.2	Mah et al., 2006
158	Peripheral blood DNA	*CSF2RA*	Xp22.32, Yp11.3	Lencz et al., 2007
479	Peripheral blood DNA	*ZNF804A*	2q32.1	O'Donovan et al., 2008
660	Peripheral blood DNA	*RELN*	7q22	Shifman et al., 2008
738	Peripheral blood DNA	*AKT1, CSF2RA, IL3RA, PRODH, RGS4, ZDHHC8, COMT, DAOA, DISC1, DRD3, DTNBP1, HTR2A, NRG1, PLXNA2, SLC6A4*	14q32.33, Xp22.33, 22q11.21, 1q23.3, 22q11.21, 13q33.2, 1q42.1, 3q13.3, 6p22.3, 13q14-q21, 8p12, 1q32.2, 17q11.2	Sullivan et al., 2008
574	Peripheral blood DNA	*CCDC60*	12q24.23	Kirov et al., 2009
119	Peripheral blood DNA	*JARID2*	6p23	Liu et al., 2009
900	Peripheral blood/saliva DNA	*ADAMTSL3*	15q25.2	Need et al., 2009
3322	Peripheral blood DNA	*MHC, MYO18B, ZNF804*	22, 6p, 22q11.2	Purcell et al., 2009
8008	Meta-analysis	*HIST1H2AG*	6p22.1	Shi et al., 2009
2663	Peripheral blood DNA	*MHC region, NRGN TCF4*	6p21, 11q24, 18q21	Stefansson et al., 2009
201	Peripheral blood DNA	*PLAA, ACSM1, ANK3*	9p21, 16p12, 10q21	Athanasiu et al., 2010
17,198	Meta-analysis	*CMYA5*	5q14.1	Chen et al., 2011
3322	Peripheral blood DNA	*NRXN1, CNTNAP2, CASK, CDC42, PRKCZ*,	2p16.3, 7q35, Xp11.4, 1p36.1, 1p36.33-p36.2	O'Dushlaine et al., 2011
1172	Meta-analysis	*ASTN2, GABR1, CNTNAP2*	9q33.1, 6q15, 7q35	Wang et al., 2010
9394	Meta-analysis	*MIR137 PCGEM1 TRIM26 CSMD1 MMP16 CNNM2 NT5C2 STT3A CCDC68, TCF4*	1p21.3, 2q32.3, 6p21.3-p22.1, 8p23.2, 8q21.3, 10q24.32, 10q24.33, 11q24.2, 18q21.2, 18q21.2	Ripke et al., 2011
18,945	Meta-analysis	*ZNF804A*	2q32.1	Williams et al., 2011
626	Peripheral blood DNA	*ELAVL2*	9p21	Yamada et al., 2011
746	Unknown	*ZKSCAN4, NKAPL, PGBD1, TSPAN18*	6p21, 6p22.1, 6p22.1, 11p11.2	Yue et al., 2011
795	Peripheral blood DNA	*SLAMF1, NFKB1, RIPK4, DOCK4, RGMB, AKAP9, CSMD1, ZCCHC14, ZNF492*	1q23.3, 4q24, 21q22.3, 7q31.1, 5q15, 7q21.2, 8p23.2, 16q24.2, 19p12	Liou et al., 2012
1169 (+2569 in the follow-up study)	Peripheral blood DNA	*AMBRA1, DGKZ, CHRM4, MDK and TCF4, CUX1*	11q1 and 18q21.2, 7q22.1^a^	Rietschel et al., 2012
5001	Peripheral blood DNA	*CACNA1C, CACNB2, TSNARE*	12p13.33, 10p12.32, 8q24.3	Ripke et al., 2013
9379	Peripheral blood DNA	*MHC, CACNA1C, MIR137, MMP16, CSMD1, STT3A*	6p21.33, 12p13.33, 1p36.22, 8q21.3, 8p23.2, 11q24.2	Cross-Disorder Group of the Psychiatric Genomics, 2013
36,989	Peripheral blood DNA and brain tissue DNA.	*DRD2, GRM3, GRIN2A, CACNA1I, GRIA1, SRR, CLCN3, RIMS1, KCTD13, NLGN4X (Selected from 108 loci)*	11q23.2, 7q21.12, 16p13.2, 22q13.1, 5q33.2, 17p13.3, 4q33, 6q12-13, 16p11.2, Xp21.33-32.	Schizophrenia Working Group of the Psychiatric Genomics, 2014
34,241 (+698)	Meta-analysis + peripheral blood DNA	*CACNA1C, CSMD1*	12p13.33, 8p23.2	Takahashi et al., 2015
1955	Meta-analysis	*NKAIN2, LSM6, GLRA1 (associated to negative symptoms presence) and KIAA1430, NRG1, PHACTR3 (associated to positive symptoms presence)*	6q22.31, 4q31.22, 5q33.1. 4q35.1, 8p12, 20q13.32 ^a^	Edwards et al., 2015

a *Genome Browser UCSC/hg38 assembly*.

Although the objective of GWAS studies is to survey the entire genome in the most systematic and unbiased way possible, and despite the optimism generated in the beginning (Sullivan and 96 Psychiatric Genetics Investigators, 2012), limits for this technique were soon clearly indicated (Pearson and Manolio, 2008) including the potential for false-positive results and genotyping errors, lack of information on gene function, the requirement for large sample sizes, and possible biases due to problems in matching cases and controls or stratification.

In addition, the extent of phenotypic variation accounted for by GWAS to date is quite low and, therefore, some have suggested that, to develop an integrative model of the relationship between genotype and clinical phenotype, we need to integrate GWAS with other functional findings that would allow a better appreciation of possible biological basis underlying the clinical characteristics of schizophrenia (Lee et al., 2012). In this sense, Wang et al. recently developed a Covariate-Modulated Mixture Model (CM3) that combine auxiliary information to GWAS data from PGC, in order to generate an “enrichment score” for each SNP. This score might help to estimate more accurately the replication probabilities for each SNP in a GWAS analysis (Wang et al., 2016).

Given that GWAS studies were unable to find a definite association of unique SNPs to schizophrenia, another option is to consider the sum of modest association of single SNP, that do not reach levels of significance, taking into account the possibility of polygenic contribution to mental illness development as proposed 50 years ago (Gottesman and Shields, 1967). This Polygenic Risk Score (PRS) is an interesting approach that summarize genetics data and risk odds ratios and was recently used as a way for stratification of schizophrenic patients, although its sensibility and specify was not enough to support its use as a predictive tool (Schizophrenia Working Group of the Psychiatric Genomics, 2014). However, some reports use PRS in order to find clinical correlations: Tesli et al., find significant association between schizophrenic or bipolar patients and its correspondent PRS (Tesli et al., 2014); Agerbo et al. also observed an association between schizophrenia and PRS in a recent meta-analysis (Agerbo et al., 2015) and, finally, Jones et al., recently reported an association between PRS and negative symptoms in adolescents (Jones et al., 2016).

Although these observations and correlations shed some light on the genetic liability to schizophrenia, the main limitation of PRS dwells in its origin as a calculated measure that do not clearly points to any specific underlying biological aspect of mental illness onset (Kendler, 2016). Also quality control of GWAS data and sample size are key for its possible use as a predictive tool, so its complete usefulness is far from established (Dudbridge, 2013).

## Quantitative trait loci (QTL)

QTL analysis is a method of localizing chromosomal regions harboring genetic variants that affect a continuously distributed polygenic phenotype (Watanabe et al., 2007), which involves the search for multiple genes each of which is neither necessary nor sufficient for the development of a specific trait. Results to date suggest linkage between different cognitive domains of schizophrenia and particular chromosomal loci 1q32.2 (D1S196), 5q (D5S111), 8p21-22 (D8S503, D8S1771), 11q23.3-24 (D11S934), 19q (D19S220), and 20q12.1-11.23 (D20S112; Silverman et al., 1996; Straub et al., 1997; Gurling et al., 2001; Sklar et al., 2004; Almasy et al., 2008) although for a disease with complex inheritance (such as schizophrenia) this type of analyses can only produce limited inferences as the nature and localization of genes related to illness susceptibility (Gurling et al., 2001).

Moreover, QTL present several problems that made more difficult the data interpretation, such as the large confidence intervals obtained from segregating populations, the difficult to distinguish two QTL that are less than 20cM apart, or the presence of too many false negatives.

As a possible solution to these disadvantages, the massive data collected from sequencing and GWAS recently allowed generating a new approach: the expression quantitative trait loci (eQTL). Unlike QTL mapping, focused in chromosomal regions that limits the number of analyzed regions, eQTL uses gene expression levels (i.e., from DNA microarrays data) as the quantitative trait (Gilad et al., 2008). The underlying assumption is if genetic expression is affected (the phenotype), a potential polymorphic marker, and probably near of the gene locus, might be responsible for this change (i.e., SNPs at regulator sequences of the gene). So, a statistical and computational approach correlates data from genetic patterns of all markers with the expression of all measured genes (Michaelson et al., 2009). With regard to schizophrenia, this approach allowed to find several new risk loci in blood samples (3p21 and 10q24 and SNPs in two calcium-channels subunits genes; Cross-Disorder Group of the Psychiatric Genomics, 2013) and also was recently used to evaluate the potential role of microRNA in its etiology (Williamson et al., 2015) or the genetic pleiotropy between immune and psychiatric disorders (Andreassen et al., 2015; Wang Q. et al., 2015).

## Endophenotypes and schizophrenia

Some authors suggest genetic analyses might be more productive if, instead of focusing on such a heterogeneous diagnostic entity, they addressed simpler biological or behavioral traits as intermediate phenotypes. These so-called “endophenotypes” have been defined as relatively simple and quantifiable biobehavioral characteristics that segregate with the illness and may suggest primary susceptibility genes that can be reliably assessed by laboratory-based measures. Based on reviews of studies in this area, it established that criteria or the selection of endophenotypes should reflect a trait characteristic of the disorder and be (1) highly heritable, indicating a robust deficit in both patients and unaffected family members; (2) rapid and easy to measure with minimal subject cooperation or effort, (3) reliable, state independent and reproducible in an individual subject, and (4) reflect an known underlying neurobiological mechanism believed relevant to the pathophysiology of the disorder and indicative of the action of a limited number of genes (Gottesman and Gould, 2003; Braff and Light, 2005; Bearden and Freimer, 2006; Turetsky et al., 2007; Braff et al., 2008; Rissling and Light, 2010; Glahn et al., 2014). The literature, as reviewed by Allen et al. (2009) reflects wealth of data on endophenotypes in schizophrenia and their first-degree relatives and very few reviews of prevalence rates within both groups and healthy controls, mainly in sensory processing and event-related potential measures, physiologic abnormalities, minor physical anomalies, measures of impaired cognitive skills, and neurobiological markers. In this report is also remarkable the aware about the normal distribution in determining endophenotypes traits, assumption that could lead to false correlations (Allen et al., 2009).

Although the endophenotype approach aims to simplify the path to understanding the biological basis of schizophrenia, its complexity is still hard to address. By way of an example, a recent association analysis of different candidate genes and schizophrenia-related endophenotypes showed extensive evidence for pleiotropy, revealing associations with three or more phenotypes and often with schizophrenia as well (see Supplementary Table 1; Greenwood et al., 2012). Further, although a recent report seems to indicate that behavioral and molecular endophenotypes could reveal heritable abnormalities in glutamatergic neurotransmission, the low sample size (34 probands with first episode psychosis, 34 first-degree relatives, and 35 unrelated healthy controls) together with a lack of replication limits inferences (Scoriels et al., 2015).

The search for valid endophenotypes, nevertheless, remains as a promising approach in filling the gap between the genetics and the development of schizophrenia. For example, the Consortium of Genetic of Schizophrenia (Gur et al., 2007) has confirmed the heritability of some traits considered as useful endophenotypes (Light et al., 2014; Seidman et al., 2015) leading the United States Food and Drug Administration (FDA) to accept demonstrated cognitive endophenotypes as therapeutic treatment targets (Braff, 2015), or the intermediate phenotypes associated to sensoriomotor function considered, by some authors, as promising intermediate phenotype for psychotic disorders (Reilly et al., 2014; Lencer et al., 2015).

## Structural and functional neuroanatomical findings

Although several structural and functional neuroimaging and post-mortem studies suggest that schizophrenia is characterized by altered neural circuits, no neuroanatomical abnormality has been clearly and consistently linked to the disorder. Despite of the controversial data obtained, these studies represent an important information source, trying to establish the mechanisms that underlie the pathophysiology of the disease. One of the earliest and most consistent findings is the ventricular enlargement in older patients with a diagnosis of schizophrenia (Andreasen et al., 1982; Nasrallah et al., 1982, 1986; DeLisi et al., 1983; Schulz et al., 1983; Reveley et al., 1984; Obiols Llandrich et al., 1986; Davis et al., 1998; Wright et al., 2000; Gaser et al., 2004; Horga et al., 2011), although a recent meta-analysis suggested that these differences could be artifacts of illness duration, age of onset, or abnormal control samples (Sayo et al., 2012). This lack of rigorous analyses could be extended to other structures, such as the prefrontal cortex, the orbitofrontal cortex, and middle frontal gyrus and structures that play an important role in the information processing, highlighting the thalamus (Lesch and Bogerts, 1984; Andreasen et al., 1990; Arciniegas et al., 1999; Konick and Friedman, 2001; Byne et al., 2002; Mileaf and Byne, 2012). On the contrary, hippocampal abnormalities are one of the main findings observed in schizophrenia patients, including changes in its volume (van Erp et al., 2015) and shape (Dean et al., 2016). Although, some reports did not find differences of hippocampal volumes between schizophrenic patients and their healthy siblings (Staal et al., 2000), reduced hippocampal volume was observed in schizophrenia but not in psychotic bipolar I disorder, leading to the authors to propose it as a differential biomarker (Arnold et al., 2015).

Regarding limbic structures, several authors suggest reduced volumes in schizophrenia including hippocampus and amygdala (Velakoulis et al., 2006), temporal gyrus (Hu et al., 2013; Guo et al., 2014) and anterior cingulate cortex (Mouchlianitis et al., 2015), findings supported by several meta-analyses (Wright et al., 2000; Arnone et al., 2009; De Peri et al., 2012).

Can these anatomical abnormalities account for the development of schizophrenia? By using functional magnetic resonance imaging (fMRI) it was observed that some brain networks show a temporal coherence, reflecting a putative functionally connection both at rest and during a task. These networks, called Intrinsic Functional Brain Networks, represent a new and interesting research field with a high potential impact for understanding the origin of mental illness (Calhoun et al., 2009). Also, changes in the Amplitude of Low-Frequency Fluctuations (ALFF), an fMRI measure associated to this spontaneous neuronal activity in specific areas of the brain, were used to investigate the underlying pathophysiology of mental disorders (Zang et al., 2007).

An even more powerful approach is to combine these data and techniques. In this sense, a combination of fMRI and gray matter (GM) volume measures in a joint-independent component analysis model, allowed to Wang et al. to discriminate healthy controls and bipolar patients from schizophrenic patients. The latter group showed higher ALFF for temporal structures, with reduced volumes of GM, suggesting that both temporal lobe function and structure might be disturbed in these patients (Wang Z. et al., 2015).

Finally, a recent report also combined electroencephalogram (EEG) data and gray matter volumes (GMV) changes. By using a joint independent component analysis, Soh et al., demonstrated that EEG oscillations (posterior alpha activity) and GMV variations (decreased volume in inferior parietal lobe, supramarginal, parahippocampal gyrus, middle frontal, inferior temporal gyri, and increased volume of uncus and culmen) might be a putative specific biomarker for schizophrenic patients (Soh et al., 2015).

All the aforementioned data shed some light about the structure-function relationship in the healthy and in the schizophrenic brain, but we also need to go deeper in order to translate these structural-functional observations to cellular processes to have a more integrated point of view for diagnosis and treatment of mental illness (Maj, 2011). In this regard, some genes previously described have been linked to altered neural circuitry characteristics of the disorder. By way of an example, rs1344706 (*ZNF804A*) is associated to increased white matter volume, which is consistent with previous reports of increased white matter volume in first-degree relatives of patients with schizophrenia (Marcelis et al., 2003), and individuals with schizotypal personality disorder (Hazlett et al., 2008). Difficulties, again, reside in replicating the results obtained. Probably we need to go beyond genetics to understand how nature interacts with nurture to produce a complex mental disease.

## Epigenetics: An introduction

Many common human diseases are influenced by a set of several genetic and environmental factors that genetics alone cannot explain (Melkonian et al., 2015). The stress-vulnerability model of etiology assumes that genetic factors operate by making individuals selectively vulnerable to environmental risks. Accordingly epigenetics refers to the interplay between environment and genes that initiate and maintain heritable patterns of gene expression and function without changing the sequence of the genome (Urdinguio et al., 2009). Like the DNA sequence, the epigenetic profile of somatic cells is preserved during mitosis but, unlike the DNA sequence which is stable and strongly conserved, epigenetic processes are highly dynamic even within an individual, being involved in the regulation of many developmental processes including the programs of gene expression that result in the development of different organs and tissues (Shipony et al., 2014).

In humans, the most widely studied epigenetic modification is the methylation of cytosine residues at the carbon 5 position (5mC) within the dinucleotide CpG (Laird, 2010) mediated by DNA methyltransferases (DNMTs), a family of enzymes that catalyze the transfer of a methyl group from S-adenosyl methionine to the DNA. These CpG dinucleotides are not randomly distributed throughout the human genome but are usually concentrated in regions called CpG islands, preferentially located at gene promoters and, although usually unmethylated in all normal tissues and mostly associated with transcriptional expression—its methylation is associated with a closed chromatin structure and transcriptional silence of the associated genes—some physiological processes require its methylation, such as the silencing of imprinted genes, the inactivation of X chromosome in females, the regulation of germline-specific genes and, finally, the silencing of tissue-specific genes in cell types in which they should not be expressed (Schubeler, 2015).

Although DNA methylation has been most widely described at CpG islands, it does not occur exclusively in these regions. First, CpG island shores, regions of lower CpG density closeness to CpG islands are associated with transcriptional inactivation by methylation. Conversely, in gene bodies DNA methylation is also common in ubiquitously expressed genes where it increases prolongation efficiency prevents spurious initiations of transcription; is positively correlated with gene expression and is also present in repetitive elements to protect chromosomal integrity by preventing the reactivation of endoparasitic sequences (Portela and Esteller, 2010), indicating the necessity to look beyond promoters, at least in human brain (Maunakea et al., 2010).

A second epigenetic mechanism is histone modification, which entail dynamic and reversible post-translational modifications of the residues at N- terminal tails of histones that are mediated by sets of enzymatic complexes that site-specifically attach or remove the corresponding chemical groups (Tessarz and Kouzarides, 2014).

The histone modifications described to date include acetylation, methylation, phosphorylation, ubiquitination, SUMOylation, and ADP-ribosylation, with a main role in processes such as DNA repair, DNA replication, alternative splicing, and chromosome condensation (Fnu et al., 2011; Park et al., 2011; Petruk et al., 2012; Zhou et al., 2012). This epigenetic mechanism has been associated with both transcriptional repression and activation and can be modified at different sites simultaneously, giving rise to cross-talk among the different markers, so its combination in a nucleosome or region plus the DNA methylation pattern specifies the outcome. In general, the acetylation of the ε-amino groups of conserved lysine residues present in histone tails due to action of histone acetylases has long been linked to a more relaxed chromatin state and, therefore, facilitates gene transcription, while histone methylation by histone methyltransferases is both associated with transcriptional activation and repression (Greer and Shi, 2012; Molina-Serrano and Kirmizis, 2013).

## Epigenetics in the human central nervous system: Brain anatomy and cognition

Dynamic relationships between DNA methylation and histone modifications reach the highest levels of complexity in the central nervous system (CNS). A great deal is known about variations in gene expression that distinguish brain regions, although the epigenetic connection to brain anatomy has not been enough explored (Ladd-Acosta et al., 2007). In this regard the epigenetic signature depends on the brain area analyzed (Ladd-Acosta et al., 2007); DNA methylation patterns vary not only from one brain region to another, but between cell types and, even, among different subpopulations of a given cell type (Iwamoto et al., 2011; Kozlenkov et al., 2014), i.e., interneurons and projection neurons. These specific epigenetic markers may help to explain brain region-specific and cell type-specific differences in gene transcription, and it could be critical to analyze the degree to which brain epigenetic signatures might be altered in disease (Ladd-Acosta et al., 2007). Moreover, recent studies that compare inter-tissue and inter-subject methylation variability reported greater correspondence of methylation patterns within a tissue across subjects than within a subject across tissues (Lokk et al., 2014; Walton et al., 2016), which indicates that the researchers interested in the epigenetic analysis of mental disorders should be careful when interpreting DNA methylation data assessed in peripheral tissues such as blood (Walton et al., 2016).

Animal models have recently shed light on a role of epigenetic mechanisms in various cognitive domains, including memory (Swank and Sweatt, 2001; Korzus et al., 2004; Oliveira et al., 2007). Early studies reported DNA methylation changes at specific gene promoters including *RELN, BDNF*, and the memory suppressor gene *PP1* in the adult hippocampus in response to fear conditioning (Levenson et al., 2006; Lubin et al., 2008; Feng et al., 2010). In addition to DNA methylation, it is well established that memory formation requires changes in histone modifications altering chromatin accessibility and the transcription of genes relevant to memory, mainly by an increase in histone acetylation (Guan et al., 2002). Conversely, a chromatin compaction that makes transcription difficult by the presence of an increase of histone deacetylases (HDAC), specifically type 2, results in a decrease of synapse number and impairs memory, whilst lower levels of this enzyme facilitate emotional memory, spatial working memory and increased synapse formation (Guan et al., 2009). Even more, a recent paper described how small RNAs can regulate memory storage in the adult brain through the epigenetic regulation of the transcription factor *CREB2* (Rajasethupathy et al., 2012) through a serotonin-dependent methylation of a conserved CpG island in the promoter region of *CREB2* that leads to enhanced long-term synaptic facilitation.

Also, the cognitive map (the spatial representation of a determined environment) could be regulated by changes in methylation patterns of place cells (hippocampal neurons that increase its activity according to specific environments). Roth et al. recently demonstrated that the methylation pattern of *Bndf*, a gene implicated in neural plasticity, differs in place cells of rats that were exposed to new environmental setups compared to a control group (rats only exposed to familiar places; Roth et al., 2015), leading to the notion that spatial experiences also alter DNA methylation patterns in specific brain regions.

Another interesting research field is focused on the epigenetic regulation of the oxytocin receptor (OXTR) levels, whether by changes in its own methylation pattern or by changes in the methylation patterns of miRNA that regulates OXTR RNA levels and its subsequent expression (Kumsta et al., 2013). In this sense, hypomethylation of miR-142 promoter and upregulation of microRNAs that target the oxytocin receptor gene was found in prefrontal cortex of patients diagnose with autism (Mor et al., 2015). It is well-known that the oxytocinergic system acts as a neuromodulator of social cognition and emotion recognition (Bukovskaya and Shmukler, 2015). In this sense, a recent report, that combines fMRI data with genetic data from blood samples, indicates that DNA methylation of the oxytocin receptor gene might predicts neural response to ambiguous social stimuli (Jack et al., 2012) and also, it was recently demonstrated that epigenetic modification of the oxytocin receptor gene could influence social cognition in humans (Puglia et al., 2015; Rubin et al., 2016). Although this is an exciting approach to epigenetic and psychological connection, the main findings with regard to methylation patterns were in blood peripheral cells, so its relevance is not clear.

## Epigenetics and disease. A focus on schizophrenia

In schizophrenia the absence of consistently replicated genetic effects together with evidence for lasting changes in gene expression after environmental exposures suggest a role of epigenetic mechanisms in its pathophysiological mechanisms (Ibi and Gonzalez-Maeso, 2015; Shorter and Miller, 2015).

The rationale for epigenetic exploration into psychiatric diseases is based in two sets of findings. First, evidence from basic research suggests that normal cognition is regulated by epigenetic mechanisms and its dysfunction occurs upon epigenetic misregulation. Second, a review by Labrie et al., suggest that epigenetic research is providing new insights into missing heritability of complex psychiatric diseases, referring to the discrepancy between epidemiological heritability and the proportion of phenotypic variation explained by DNA sequence differences (Labrie et al., 2012).

**Figure 1:**
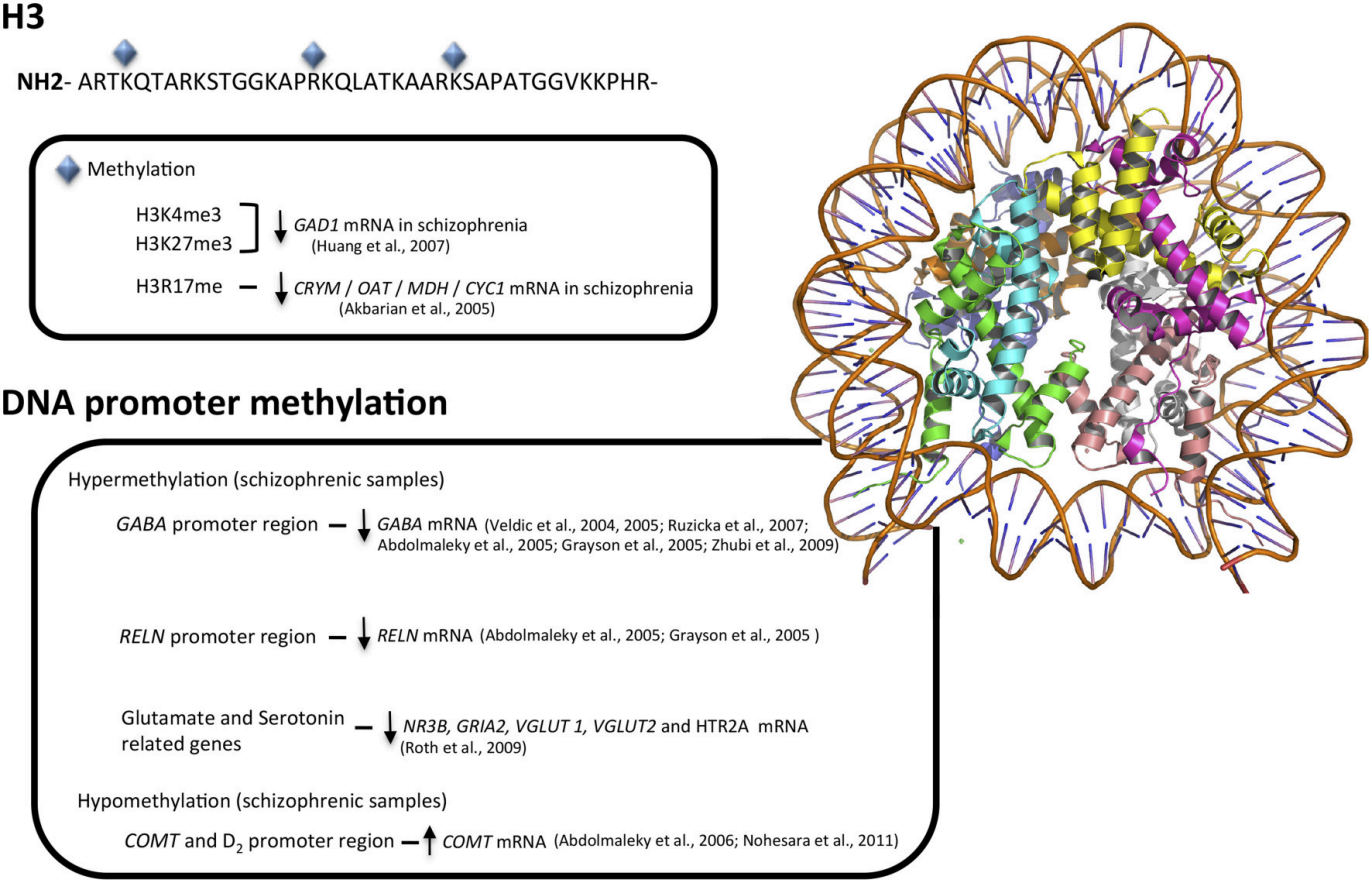
**Major epigenetic findings in schizophrenia**. Above, histone modifications that result in euchromatin or heterochromatin states, allowing transcription factors access to genes or blocking transcription at specific loci. Below, hyper- and hypo-methylation patterns associated with schizophrenia in GABA, glutamate, serotonin, and dopamine neurotransmitter systems.

To date, most studies exploring epigenetic mechanisms in schizophrenia have employed post-mortem human brain samples. Pioneering studies have focused on different genes that have been related with the pathophysiology of the disease, including differences in the amount of S-adenosyl methionine (Guidotti et al., 2007) or an overexpression of HDAC1 in the prefrontal cortex of patients with schizophrenia (Sharma et al., 2008). Other studies reported an increase in DNMT mRNA and protein levels in the cortical GABAergic system of individuals with schizophrenia (Veldic et al., 2004, 2005; Ruzicka et al., 2007; Zhubi et al., 2009; Figure 1), suggesting that the down-regulation of GABAergic transcripts is due to hypermethylation of their gene promoters (Abdolmaleky et al., 2005; Grayson et al., 2005), results not confirmed by the pyrosequencing method in a later study (Tochigi et al., 2008).

In addition to the GABAergic system, epigenetic modifications in schizophrenia have been described in serotoninergic (Abdolmaleky et al., 2011, 2014; Carrard et al., 2011; Ghadirivasfi et al., 2011), dopaminergic (Abdolmaleky et al., 2006), and glutamatergic systems (Mill et al., 2008) reporting altered CpG methylation of glutamate receptors genes *NR3B* and *GRIA2*, glutamate transporters *VGLUT1* and *2* and the serotoninergic receptor *HTR2A*.

Regarding the *MB-COMT* promoter, an unmethylated pattern that correlates with hypomethylation of the dopamine D2 receptor gene has been described in the schizophrenic frontal lobe (Abdolmaleky et al., 2006) and in saliva (Nohesara et al., 2011), changes not replicated in an independent study (Mill et al., 2008). A brief summary of these findings is showed in Figure 1.

The failure of replication could be due to a number of factors, including the considerable heterogeneity of the DNA methylation patterns between individuals (Kaminsky et al., 2009) or even the use of tissue homogenate that is comprised of an extremely heterogeneous mixture of different cell types (Akbarian, 2010). Besides inter-individual differences, other factors could influence on DNA methyltransferases, changing the methylation pattern of different genes, such as the social environment (Rampon et al., 2000; Weaver et al., 2004), the environmental toxins (Desaulniers et al., 2005; Bollati et al., 2007) or antipsychotic drugs (Shimabukuro et al., 2006; Cheng et al., 2008; Abdolmaleky et al., 2015); by way of an example, the administration in clinically relevant doses of different antipsychotics in mice, specifically clozapine and sulpiride in association or not with valproate, down-regulate *Reln* and *Gad1* promoter methylation in the frontal cortex and striatum (Dong et al., 2008).

Lastly, we found that several thousand individual CpGs demonstrated small, but statistically significant, differences in DNAm levels between adult patients with schizophrenia and controls that did not appear confounded by cellular composition or smoking. The differences found between patients and controls appear to represent epigenetic marks that principally associate with early neurodevelopment and not with events that herald the onset of the disorder or that characterize adult brain biology. Overall, the data suggest that both the genetic and environmental risk components of schizophrenia involve early developmental influences.

Up to now, we focused on specific marks at specific genes but what do we know about the distribution of aberrant DNA methylation in the human genome? Although it remains very superficially and inadequately studied (Schumacher et al., 2006), the development of new technology makes it possible to carry out epigenome-wide association studies (EWAS) to analyze the DNA methylation status of a great number of CpG, (i.e., 450.000 methylation sites per sample at single-nucleotide resolution), which is directly comparable to highly successful GWAS chips.

As an example of the usefulness of this approach, a recent study carried out by Jaffe et al. characterized the methylation pattern in prefrontal cortex in 335 healthy controls and 191 patients with schizophrenia. This research has demonstrated that shifts in neuronal composition across lifespan are associated to changes in DNA methylation patterns and by assessing 485,000 sequences of the epigenome, authors found that these changes were mainly concentrated in genomic regions that might confer clinical risk for schizophrenia development (Jaffe et al., 2016). Although this work does not clearly reflect a specific methylation signature for schizophrenia development, it shed some light about the role of epigenetic changes as an intermediate for mental illness onset. In this regard, the increasing body of data obtained by applying this approach with post-mortem brain tissue and whole blood DNA suggest several genes that could be associated with different aspects of the pathophysiology of the disease (Mill et al., 2008; Dempster et al., 2011; Kinoshita et al., 2013). On Table 2, we collect a brief summary of the main findings obtained by applying the EWAS approach in order to study the schizophrenic brain.

**Table 2:** **Epigenome-wide association studies in schizophrenia**.

**Number of schizophrenic samples**	**Type of samples**	**Genes of interest**	**Chromosome/Locus**	**References**
35	Frontal cortex post-mortem brain tissue	*AUTS2, GRIA2, GLS2, HELT, HCG9, LHX5, LMX1B, JAKMIP1, NR4A2, PLA2G4B, GIRK2, RAI1, SLC17A6, SLC17A7, WD* Repeat Domain 18	7q11.22, 4q31.1, 12q13.2, 4q35.1, 6p21.33, 12q24.13, 9q33.3, 4p16.1, 2q24.1, 15q15.1, 21q22.13, 17p11.2, 11p14.3, 19q13.33, 19p13.3	Mill et al., 2008
44 (monozygotic twins)	Peripheral blood DNA	*PUS3, SYNGR2, KDELR1, PDK3, PPARGC1A, ACADL, FLJ90650, TUBB6*	11q24.2, 17q25.3, 19q13.3, Xp22.11, 4p15.1, 2q34, 5q23.1, 18p11.21	Dempster et al., 2011
30 (6 monozygotic twins)	Peripheral blood DNA	*PPP1R13L, PVRL4, CRTAP, HSPA1B, DEFB123*	19q13.32, 1q23.3, 3p22.3, 6p21.3, 20q11.1	Kinoshita et al., 2013
18	Peripheral blood DNA	*HTR1E, COMTD1, ADAMTS3*	6q14.3, 10q22.2, 4q13.3 ^a^	Nishioka et al., 2013
63	Peripheral blood DNA	Of 2552 CpG sites, 1161 (45.5%) demonstrated higher DNA methylation		Kinoshita et al., 2014
24	Frontal cortex post-mortem brain tissue	*NOS1, AKT1, DNMT1, SOX10, DTNBP1 and PPP3CC*	12q24.22, 14q32.33, 19p13.2, 22q13.1, 6p22.3, 8p21.3 ^a^	Wockner et al., 2014
39	Cerebellum	*PIK3R1, BTN3A3, NHLH1, SLC16A7*	5q13.1, 6p22.2, 1q23.2, 12q14.1	Chen et al., 2014
20/21	Frontal cortex post-mortem brain tissue / Cerebellum	*GSDMD, RASA3, HTR5A, PPFIA1*	8q24.3, 13q34, 7q36.2, 11q13.3 ^a^	Pidsley et al., 2014
2 (2 female monozygotic twins)	Peripheral blood DNA	*A) DGKI, DISC1, DRD3, DTNBP1, FXR1, GRIA1, GRIN2B (among 58 other genes for twins from family 1) B) DAOA, DGKI, DISC1, DRD3, IMMP2L, NRG1 (among 13other genes for twins from family 2)*	7q33, 1q42.2, 3q13.31, 6p22.3, 3q26.33, 5q33.2, 12p13.1 13q33.2, 7q31.1, 8p12	Castellani et al., 2015

a *Genome Browser UCSC/hg38 assembly*.

The main second epigenetic mechanism, histone modification, has been less explored in schizophrenia, both in peripheral blood cells or post-mortem brain tissue. Focusing on the distribution of two histone markers, H3K4me3 and H3K27me3, a shift has been reported in prefrontal cortex in the chromatin surrounding *GAD1* promoter that is accompanied by a decrease in *GAD1* mRNA (Huang and Akbarian, 2007). A more recent report also demonstrated that, in fact, the complete 3D chromosomal structure (heavily influenced by its histones marks) is necessary to allow the correct regulation of this gene (Bharadwaj et al., 2013). Reinforcing the role of chromatin structure and specific histone marks, the mRNA levels of the highly related isoform GAD2 in prefrontal cortex were similar between schizophrenic patients and their healthy relative (Glausier et al., 2015).

A third histone marker, the methylation of histone H3 at arginine 17, a marker of closed chromatin state and, therefore, transcriptional repression, has been involved in the down-regulation in schizophrenia of several metabolic genes such as *CRYM, OAT, MDH*, and *CYC1* (Akbarian et al., 2005; Figure 1). Also, higher mRNA levels of *G9a, GLP*, and *SETDB1* (three histone methyltransferases that catalyzes the methylation of H3 at lysine 9, a well-known epigenetic repressive mark) were observed in brain samples of schizophrenic patients compare to healthy controls. In fact, levels of G9a mRNA were significantly correlated with increased negative subscales scores on the PANSS (Positive and Negative Syndrome Scale; Chase et al., 2013).

Finally, Kurita and colleagues found a relationship between long treatment with antipsychotics and down-regulation of *GRM2*, a metabotropic glutamate 2 receptor, through decreased histone acetylation at its promoter region in the human frontal cortex, which could represent a promising new target for schizophrenia treatment (Kurita et al., 2012).

## Future directions

The study of the epigenetic mechanisms at defined gene regions in schizophrenia samples represents a new approach that could potentially uncover molecular mechanisms of deregulated gene expression in this complex disorder. As discussed above, crosstalk between the different epigenetic markers could explain some aspects of schizophrenia pathophysiology but, at the same time, present a complex picture characterized by instability of the epigenetic code that could be interpreted as a double-edge sword, that is presenting a plausible mechanism but one that, by virtue of its complexity is exceedingly difficult to study. Unanswered questions remains as to whether epimutations reflect long-lasting and sustained defects in the regulation of gene expression (Akbarian, 2010) as well as the effects of their genomic distribution and/or tissue specificity. Of course, we must carefully take into account the influence of the sample size, sample type and epigenomic assay needed to reliably detect disease-associated epimutations (Labrie et al., 2012) in clinical samples.

As we have pointed out, the link between the molecular basis of schizophrenia and clinical features remains undefined. To date, neurobiology of the disorder does not match with its clinical classification, may be due to the fact that different biological pathways that lead to schizophrenic symptoms could occur simultaneously as independent or as interdependent processes, as it has been proposed by Maric and Svrakic (2012); for example, down-regulation of telencephalic GABAergic and/or NMDA receptors genes might accounts for several structural and functional alterations that could underlie schizophrenic symptoms (Farber, 2003; Lisman et al., 2008). Although the non-linear nature of schizophrenia supposes that its clinical expression and evolution are highly variable among patients, to look for a link between clinical aspects and molecular biology it could be fruitful to focus on the study of epigenetic markers in non-affected siblings of patients with a diagnosis of schizophrenia (van Os et al., 2010).

A third challenge for identification of specific epimutations in schizophrenia is to clarify when an epigenetic alteration is casual or if it is a consequence of the disease. Some authors suggest that identifying the epimutation in multiple tissues of schizophrenic patients in the germ line of affected individuals or their fathers, would favor but not prove a causal relationship (Glatt et al., 2011), although it has been suggested that the relevance of findings from of DNA isolated from peripheral blood samples to brain processes and their relevance as potential biomarkers for neuropsychiatric disorders is also questionable (Davies et al., 2012).

The recent description of different methylation patterns at different CpG sites in the normal human brain highlights that gene regulation mechanisms in the CNS are highly complex. In this way, technological trends for epigenetic assays have shown a great advance that allows researchers to analyze from selected loci to the complete epigenome using continually smaller starting populations of cells; for example EWAS approaches could address that complexity giving us a complete panoramic picture of the DNA methylation patterns in an specific tissue or in a cell population; moreover, recently it has been assigned new functions to the intergenic sequences, acting as DNA marks for proteins that could influence gene activity (Pennisi, 2012). This approach could enhance our understanding of the gene regulation processes in the human brain, which are heterogeneous across cell types and show different epigenetic signatures depending on the brain area analyzed (Ladd-Acosta et al., 2007) and, therefore, its combination with GWAS data in brain studies with relatively little-studied histone modifications could uncover genetic-epigenetic interactions in schizophrenia and establish predisposition factors to this complex psychiatric disorder.

**Figure 2:**
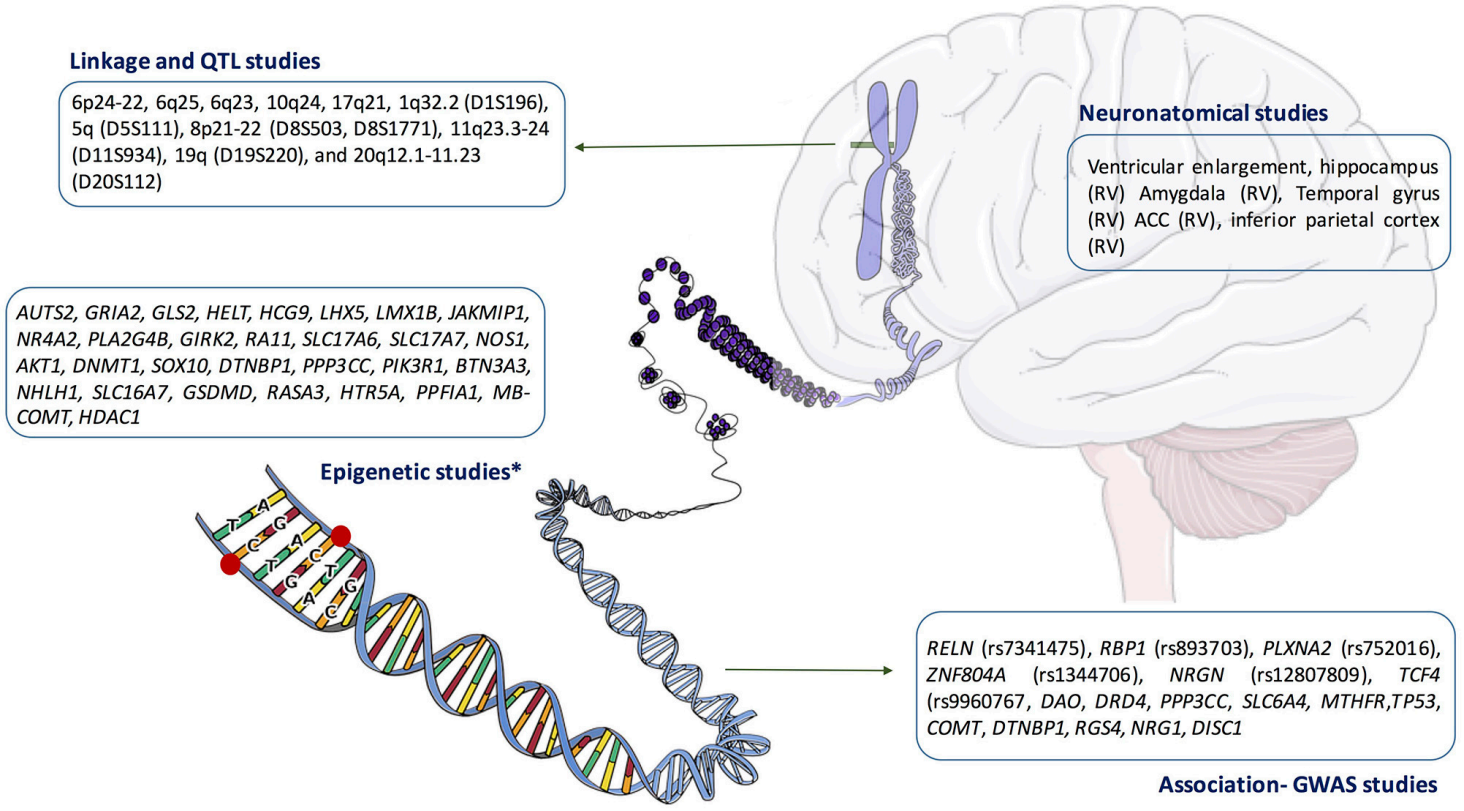
**Summary of the main findings in the neurobiology of schizophrenia**. ACC, anterior cingulate cortex; RV, reduce volume. ^*^Main epigenetic findings in human brain samples.

In summary, as we have pointed before, there are many findings that have been presented as potential biomarkers for schizophrenia, including data from linkage, association, neuroanatomical, or genetic studies (a brief summary of these findings is showed in Figure 2). This mass of data, characterized by its absence of reliability and validity, is increasingly seen as a sign of uncertainty and confusion (Maj, 2011). In this respect, we want not contribute to this chaos, and therefore, we will simply point out that epigenetic studies may shed light on the complex interaction that occurs between nature and genetics to produce a mental illness; perhaps this is not sufficient to understand (in terms of Jaspers) what schizophrenia is, but could help us define some variables that may contribute to its onset and/or development. However, we think it is important to start from the beginning and therefore it is necessary to review the current diagnostic criteria for mental disease which, in general, and schizophrenia in particular, define a disorder by what it is not rather by what it is, which involves a recognized limitation for this approach. In agreed with Sullivan et al., we also need to keep in mind that, in psychiatric, we are at the end of the beginning, not the beginning of the end and we will need more scientific cooperation, a more clever research strategy, and higher statistical rigor in order to get a complete picture of schizophrenia neurobiology (Sullivan et al., 2012).

## Author contributions

AC, JS, RA: acquired, analyzed and interpreted data for the work. This also included, drafting the work or revising it critically for important intellectual content. AC, JS, RA: Agreement to be accountable for all aspects of the work in ensuring that questions related to the accuracy or integrity of any part of the work are appropriately investigated and resolved. AC, JS, RA: Final approval of the version to be submitted.

### Conflict of interest statement

The authors declare that the research was conducted in the absence of any commercial or financial relationships that could be construed as a potential conflict of interest.
