# Supplementary material for: From Linkage Studies to Epigenetics: What We Know and What We Need to Know in the Neurobiology of Schizophrenia
Source: Front Neurosci. 2016 May 11;10:202. doi: 10.3389/fnins.2016.00202 (PMC4862989; doi:10.3389/fnins.2016.00202)
Supplement: Supplementary file 1 [file Table1.docx]

| **Gene** | **Location** | **Phenotypes** | **Association with schizophrenia** |
| --- | --- | --- | --- |
| *GRIK3* | *1p34.3* | Prepulse inhibition; CVLT-II immediate recall; WCST-64 perseverative responses | NO |
| *NOS1AP* | *1q23.3* | Antisaccade; Startle habituation; CVLT-II delayed recall | NO |
| *CTNNA2* | *2p12* | Prepulse inhibition; P50 S1 amplitude; LNS working memory; CVLT-II immediate recall; WCST-64 categories completed | NO |
| *ERBB4* | *2q34* | LNS immediate recall; CVLT-II immediate recall; CVLT-II delayed recall; WCST-64 categories completed | YES |
| *GRID2* | *4q22.3* | Prepulse inhibition; Startle habituation; P50 S1 amplitude; LNS immediate recall; CVLT-II immediate recall; WCST-64 perseverative responses | YES |
| *RELN* | *7q22.1* | P50 S1 amplitude; CVLT-II delayed recall; WCST-64 categories completed | YES |
| *NRG1* | *8p12* | Startle habituation, P50 S1 amplitude; LNS immediate recall; WCST-64 perseverative responses; WCST-64 categories completed | YES |
| *GRIK4* | *11q23.3* | LNS immediate recall; CVLT-II immediate recall; CVLT-II delayed recall | NO |
| *GRIN2B* | *12p13.1* | Startle habituation; LNS working memory; CVLT-II immediate recall | YES |
| *CHRNA7* | *15q13.3* | Antisaccade; CVLT-II delayed recall; WCST-64 perseverative responses | NO |
| *CACNG2* | *22q12.3* | Antisaccade; CVLT-II immediate recall; CVLT-II delayed recall; WCST-64 categories completed | NO |

Supplementary Table 1. Summary of the genes that display extensive evidence for pleiotropy ([Greenwood et al., 2012](#_ENREF_53)).
